# Berberine Protects Brain Endothelial Barrier via β-Catenin: A Network Pharmacology in Pigs and In Vitro Mechanistic Analysis

**DOI:** 10.3390/ani16203240

**Published:** 2026-10-15

**Authors:** Pu Guo, Yangfan Dong, Yu Cheng, Yunda Xue, Qirong Lu, Zhongyuan Wu, Chun Ye, Yu Liu, Yinsheng Qiu

**Affiliations:** 1 Hubei Key Laboratory of Animal Nutrition and Feed Science, Wuhan Polytechnic University, Wuhan 430023, China; guopu@whpu.edu.cn (P.G.); 13297953032@163.com (Y.D.); 13246187826@163.com (Y.C.); 15823132897@163.com (Y.X.); qirongluvet@whpu.edu.cn (Q.L.); zhongywu@whpu.edu.cn (Z.W.); yechun@whpu.edu.cn (C.Y.); 2 Wuhan Engineering and Technology Research Center of Animal Disease-Resistant Nutrition, School of Animal Science and Nutritional Engineering, Wuhan Polytechnic University, Wuhan 430023, China

**Keywords:** *Glaesserella parasuis*, berberine, CTNNB1, network pharmacology, meningitis

## Abstract

*Glaesserella parasuis* (*G. parasuis*) is an important swine pathogen that causes Glässer’s disease, of which meningitis is among the most severe clinical symptoms. However, the molecular mechanisms underlying *G. parasuis*-induced meningitis remain poorly understood. In this study, we employed a network pharmacology-based strategy combined with in vitro experimental validation to investigate the protective effects and mechanisms of berberine against *G. parasuis* meningitis. Network pharmacology analysis identified CTNNB1 (encodes the β-catenin protein) as a core target among multiple candidate targets. Gene Ontology (GO) and Kyoto Encyclopedia of Genes and Genomes (KEGG) enrichment analyses revealed that the predicted targets of berberine are significantly associated with inflammatory signaling (mitogen-activated protein kinase MAPK/nuclear factor kappa B NF-κB) and stress-response pathways (forkhead box O FoxO/p53), suggesting a multifaceted protective mechanism against *G. parasuis*-induced blood–brain barrier (BBB) disruption. Molecular docking results combined with drug affinity responsive target stability (DARTS) assays demonstrated that berberine binds directly to β-catenin with high affinity (docking score = −7.52 kcal/mol), as evidenced by enhanced proteolytic stability of β-catenin upon berberine (2.5, 10, and 20 μg/mL) treatment. To further validate the functional relevance, we introduced zonula occludens-1 (ZO-1), a representative tight junction protein, as a functional verification indicator. Our results demonstrated that *G. parasuis* infection significantly downregulated the expression of both ZO-1 and β-catenin in human brain microvascular endothelial cells (hBMECs), while berberine (2.5, 10, and 20 μg/mL) treatment effectively reversed this downregulation in a concentration-dependent manner. These findings reveal that berberine exerts its protective effects against *G. parasuis* meningitis by targeting CTNNB1 to restore intercellular junctional integrity—including tight junctions (ZO-1) and adherens junctions (β-catenin)—thereby preserving BBB function. This study provides a mechanistic basis for the development of berberine as a promising alternative therapeutic agent against *G. parasuis* meningitis.

## Simple Summary

Meningitis caused by *Glaesserella parasuis* (*G. parasuis*) is easily overlooked and poorly understood. Combining network pharmacology with in vitro experiments, we show that berberine directly targets CTNNB1 (β-catenin) and modulates inflammatory and stress response pathways, highlighting its potential as an antibiotic alternative for *G. parasuis* meningitis.

## 1. Introduction

*Glaesserella parasuis* (*G. parasuis*), a gram-negative bacterium belonging to the family *Pasteurellaceae,* is one of the most important bacterial swine pathogens [1,2]. This bacterium normally exists in the porcine respiratory tract, causing Glässer’s disease and pneumonia in pigs. The clinical signs of this disease include polyarthritis, fibrinous polyserositis, and meningitis, resulting in substantial economic losses to the global swine industry [3,4,5]. The symptoms of meningitis, often overlooked, have drawn growing attention in recent years as the number of reported cases continues to rise. In this context, advanced omics approaches have been employed to unravel the underlying mechanisms. Guo et al. [6] performed an integrative analysis of the DNA methylome and transcriptome in porcine brain tissue infected with *G. parasuis*, identifying numerous differentially methylated regions and genes; however, the functional relevance of these molecular changes and their potential as therapeutic targets remain to be elucidated. Meanwhile, another study investigated the role of the PI3K/Akt/Erk signaling pathway in a mouse model of meningitis induced by *G. parasuis* and observed that quercetin modulated this pathway [7], but the precise molecular targets and the protective mechanisms against blood–brain barrier (BBB) disruption are still largely unknown, warranting further investigation. Collectively, these studies underscore the ongoing efforts to decipher the pathogenesis of this neglected disease, yet the identification of definitive molecular targets and effective therapeutic strategies remains a critical unmet need.

Although 15 serovars of *G. parasuis* have been formally defined, more than 20% of clinical isolates remain unserotyped [2,8]. This serovar diversity, together with the uncertainty in serotyping, poses a major obstacle to the effective prevention and control of Glässer’s disease. Control of Glässer’s disease relies on antimicrobial therapy and vaccination, yet each has limitations. Antimicrobials are commonly used, yet resistance is a growing concern. Indeed, regional surveillance data have revealed marked variations in resistance rates: for β-lactams, resistance was below 10% in England and Spain, but reached 30% and 50% in Hainan and Zhejiang, respectively; for tetracyclines, resistance was 6.7% in the United Kingdom, exceeded 40% in Spain, and was approximately 33% in Hainan and 75% in Zhejiang [9,10]. Furthermore, over 50% of isolates collected during 2014–2017 were resistant to flumequine and neomycin, and antimicrobial use may disrupt colonization and immunity; thus, their use is advised only in limited outbreak situations [11]. Commercial inactivated vaccines are largely serovar-specific with limited cross-protection, and even within the same serovar, strain antigenic differences can result in incomplete protection [12]. Collectively, these findings underscore the growing threat of antimicrobial resistance, prompting an urgent need for the development of alternative therapeutic strategies—such as safe and effective natural products that could modulate microbiota, enhance immunity, or directly inhibit the pathogen—against this pathogen.

Meningitis represents one of the most severe manifestations of *G. parasuis* infection, where the above-mentioned limitations of conventional control measures become particularly critical [13]. Antimicrobial therapy and vaccination remain the mainstays of Glässer’s disease control, but treating central nervous system infections poses particular challenges, as the BBB limits many antimicrobial agents from entering the brain [14]. Moreover, the neuroinflammation triggered by bacteria crossing the BBB can cause irreversible tissue damage and worsen the prognosis [15,16]. Given the global limitations of existing control measures—herd management, antimicrobial therapy, and vaccination—and the growing threat of antimicrobial resistance, the development of natural drugs or novel delivery strategies capable of effectively crossing the BBB for *G. parasuis* meningitis is particularly urgent.

Berberine, a quaternary ammonium isoquinoline alkaloid widely found in various plants, has a long history of medicinal use in China and exhibits diverse pharmacological activities, including anti-inflammatory, antibacterial, neuroprotective, and anti-atherosclerotic effects [17,18]. Berberine exerts cardiovascular protective effects by regulating the synthesis and secretion of neurotransmitters in the central nervous system, and has shown potent efficacy against various neurological diseases, including stroke, depression, and Alzheimer’s disease [19,20,21,22]. Moreover, previous research has indicated that berberine is able to pass through the BBB and thereby produce its therapeutic effects [23]. Berberine has also been demonstrated to exert therapeutic effects against *G. parasuis* infection in vivo, offering significant protection against *G. parasuis* infection in mice, with protective efficacy comparable to that of ceftazidime under experimental conditions, and with an in vitro minimum inhibitory concentration (MIC) of 2 μg/mL [24]. However, the molecular mechanism by which berberine exerts its protective effects against *G. parasuis*-induced meningitis remains elusive.

In the present study, we employed a network pharmacology-based strategy, combined with molecular docking and in vitro validation in human brain microvascular endothelial cells (hBMECs), to systematically investigate the active ingredients, potential targets, and underlying mechanisms of berberine against *G. parasuis*-induced meningitis, with a particular focus on its protective role in maintaining BBB integrity through the modulation of junctional proteins. This study aims to provide a theoretical basis and experimental foundation for the development of novel therapeutic agents against this challenging infection.

## 2. Materials and Methods

### 2.1. Chemicals and Reagents

The *G. parasuis* SH0165 strains used were preserved in our laboratory. hBMECs were provided by Professor Xu Wang, College of Veterinary Medicine, Huazhong Agricultural University, Wuhan, Hubei, China. Berberine (≥98%) was purchased from Chengdu Biopurify Phytochemicals Ltd. (Chengdu, China). The GAPDH antibody (GB11002) was purchased from Wuhan Servicebio Technology Co., Ltd. (Wuhan, China). The β-catenin antibody (PTM-6919) was purchased from PTM BIO LLC (Hangzhou, China). The ZO-1 antibody (A0659) was purchased from ABclonal Biotechnology Co., Ltd. (Wuhan, China).

### 2.2. Network Pharmacological Analysis

Berberine targets were identified using the following three databases: PharmMapper, SwissTargetPrediction, and the Traditional Chinese Medicine Systems Pharmacology Database and Analysis Platform [23]. The protein targets of meningitis were obtained from GeneCard (https://auth.lifemapsc.com/). The PubMed database was searched with the keywords ‘*Glaesserella parasuis*’ to find the bacterial targets. Duplicates were removed to obtain a list of potential disease targets. To further identify the core targets, Venn analysis and protein–protein interaction (PPI) network analysis were performed using an online Venn diagram tool (www.bioinformatics.com.cn) and the STRING database (https://cn.string-db.org), respectively. Then, the PPI results were imported into Cytoscape 3.7.2 software [25] for topological analysis. Genes were sorted according to the degree values, and those with scores greater than the average were selected as the core targets. Moreover, GO and KEGG enrichment analysis was performed for the common targets. The online DAVID database (https://davidbioinformatics.nih.gov) was used for GO and KEGG enrichment analysis, with the species set to *S. scrofa* and the *p*-value set at <0.05. The enrichment results were processed using bioinformatics (http://www.bioinformatics.com.cn/) to obtain histograms and bubble charts.

### 2.3. Molecular Docking and DARTS Coupled

The structure of the active pharmaceutical ingredient was downloaded from the PubChem database and imported into the ChemBio3D software (version 25.0) for processing; the energy was minimized, and it was saved as a ligand. In the UniProt database (https://www.uniprot.org), the species was set to *S. scrofa*, and the 3D structure of the target protein was exported. Protein-ligand interaction profiler (PLIP version 2.4) [26] was used to simulate the ligand-receptor docking.

Lysates from hBMECs were incubated with or without berberine (2.5, 10, and 20 μg/mL) for 9 h at room temperature, and lysates were then divided into seven portions for digestion with 1:8000 pronase E (1074330001, Sigma-Aldrich, Shanghai, China) for 20 min at room temperature, as described by Geng et al. [27]. Then the digestion was stopped by adding protease inhibitors, and the samples were used for WB analysis.

### 2.4. Bacterial Growth Curve and hBMECs Infected with G. Parasuis

*G. parasuis* was inoculated into TSB (with serum and NAD+) liquid medium and incubated in a constant temperature shaker at 37 °C, 180 r/min for about 24 h. Under aseptic conditions, 100 μL of *G. parasuis* was inoculated into TSB (with serum and NAD+), and the TSB (with serum and NAD+) liquid medium was incubated at 37 °C, 180 r/min, at constant temperature. The growth curve was determined by measuring absorbance (OD = 600 nm) at 2 h, 4 h, 6 h, 8 h, 10 h, 12 h, and 24 h using a UV spectrophotometer (MAPADA, Shanghai, China). Meanwhile, at the above time points, the bacterial solution was diluted in a gradient to carry out colony counting. The growth curve of *G. parasuis* was run in triplicate and drawn based on the results of OD600 and colony counting.

hBMECs were spread on plates and counted when the cell growth density reached 70%. *G. parasuis* was added to the cells at different ratios, and photographs were taken at different time points to observe the survival of hBMECs, while a TSB broth (with serum and NAD+) without bacterial suspension was used as a negative control.

### 2.5. Cell Culture and Cell Viability Assay

hBMECs were grown in incubators at 37 °C and 5% CO_2_. Proliferating cells were cultured in RPMI1640 medium supplemented with 10% FBS, 2 mM L-glutamine, 1 mM sodium pyruvate, nonessential amino acids, vitamins, and penicillin and streptomycin (100 U/mL).

Cell viability was determined using a CCK-8 assay. hBMECs were seeded into 96-well plates for 24 h, then incubated with a series of berberine concentrations (2.5 μg/mL −160 μg/mL) for 24 h. Then, 10 μL of CCK-8 solution (Vazyme, Nanjing, China) was added to each well of the plate; the plate was incubated for 1–4 h, and the absorbance was measured at 450 nm using a microplate reader (Spectra Max i3x, Molecular Devices, Shanghai, China). The cell viability was calculated according to the formula in the product manual.

### 2.6. Immunofluorescence Analysis

hBMECs were inoculated onto slides of 12-well plates, and the medium was discarded after berberine (2.5, 10, and 20 μg/mL) and *G. parasuis* (MOI = 10) treatment. The cells were fixed with 4% paraformaldehyde at room temperature for 30 min, washed 3 times with 0.02 M phosphate-buffered saline (PBS) at room temperature for 3 min, and incubated with fixative solution (4% formaldehyde) at room temperature for 15 min. Anti-ZO-1 and anti-CTNNB1 primary antibody dilutions were added to the cells and incubated at 4 °C overnight. Cells were washed with PBS, incubated with secondary antibody (mixture including ABflo^®^ 488-conjugated Goat Anti-Rabbit IgG and Cy3 Goat Anti-Mouse IgG, ABclonal, Wuhan, China) for 1 h at 37 °C in the dark (goat anti-rabbit 488 and goat anti-mouse cy3, ABclonal, China), and washed 3 times with PBS at room temperature for 10 min. Then, the cells were incubated in DAPI (5 μM, Invitrogen Eugene, OR, USA) staining solution for 10 min and washed in TBS to monitor cellular fluorescence with an UltraVIEW VOX Confocal system (PerkinElmer, Co., Norwalk, CT, USA) using a 60×, 1.4 NA oil immersion objective lens.

### 2.7. Statistical Analysis

The experimental data were expressed as the mean ± SD. The difference between two groups was analyzed using the two-tailed Student’s *t*-test. Compared with the control group, ** = *p* <0.01 indicates a highly significant difference.

## 3. Results

### 3.1. Berberine and the Identification of Core Targets for G. parasuis Meningitis

**Figure 1:**
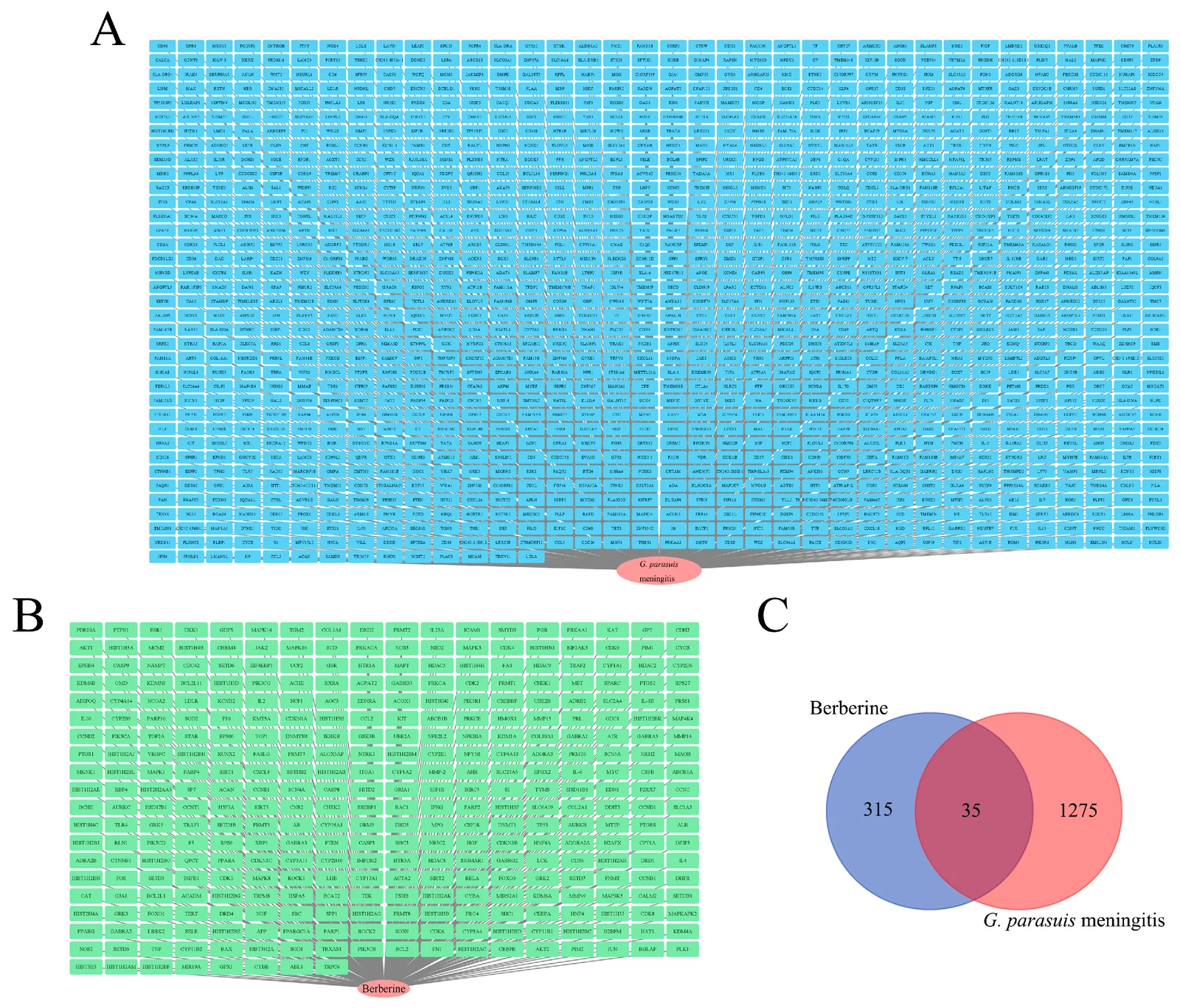
Berberine and *G. parasuis* meningitis targets. (**A**) *G. parasuis* meningitis targets. (**B**) Berberine targets. (**C**) Venn diagram of the two groups of targets.

Network pharmacology combined with bioinformatics was used to mine the targets of berberine to alleviate *G. parasuis* meningitis. The berberine, *G. parasuis*, and meningitis targets are summarized in Figure 1. We identified 350 berberine targets (Figure 1A) and 1310 *G. parasuis* meningitis targets (Figure 1B).

**Figure 2:**
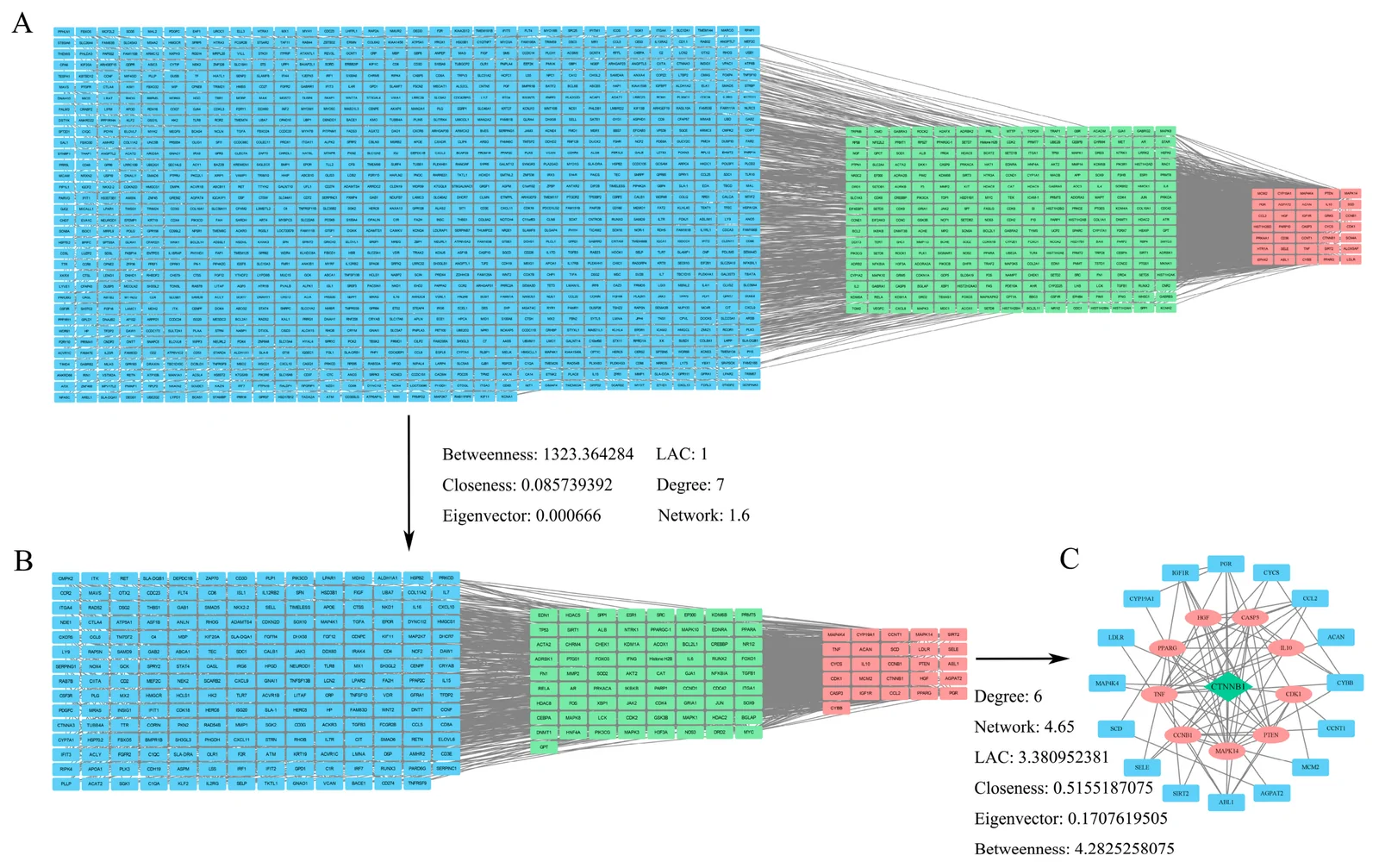
Screening of the core targets of berberine in alleviating *G. parasuis* meningitis based on the STRING database and Cytoscape. (**A**) Cytoscape analysis of the interconnection of berberine and *G. parasuis* meningitis targets (Note: blue represents *G. parasuis* meningitis targets; green represents berberine targets). (**B**) Cytoscape parameters (betweenness > 1323.364284, closeness > 0.085739392) were set to evaluate the relationship between the key targets (Note: blue represents *G. parasuis* meningitis targets; green represents berberine targets). (**C**) core targets for further screening based on the Cytoscape database.

There are 35 common targets between berberine and *G. parasuis* meningitis targets in a Venn diagram (Figure 1C). These 35 potential therapeutic targets were processed at the STRING online analysis site to map the protein–protein interaction network (PPI) (Figure 2A). We submitted the target proteins to STRING version 11.0 (http://string-db.org/) to construct a PPI network, which indicates 26 core targets (Figure 2C). Among them, CTNNB1 (encodes the β-catenin protein) is the most critical target, and it could be a berberine and *G. parasuis* meningitis target.

### 3.2. GO Enrichment and KEGG Pathway Analysis of Core PPI Network

**Figure 3:**
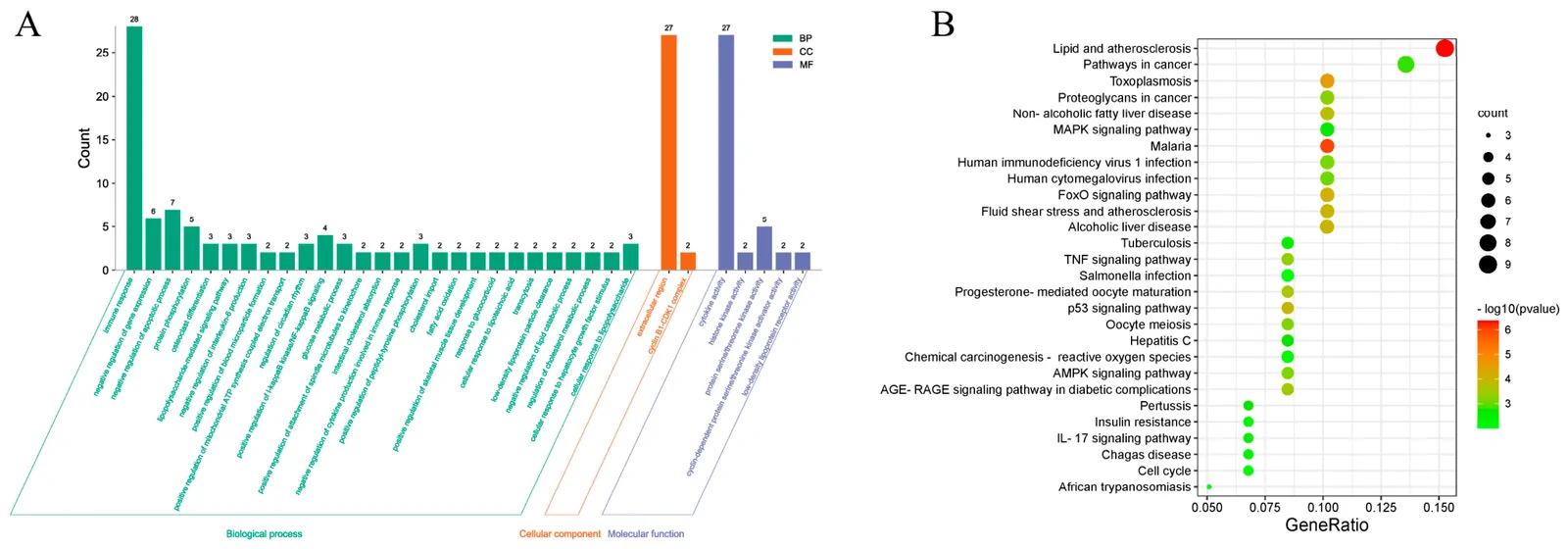
(**A**) GO and (**B**) KEGG enrichment analysis of potential targets of berberine to alleviate *G. parasuis* meningitis.

To further elucidate the potential roles of core targets in gene function and signal transduction pathways, GO enrichment analysis and KEGG pathway enrichment analysis were performed using the DAVID database. The GO analysis covered three functional categories—biological process, cellular component, and molecular function—and a total of 34 GO terms were significantly enriched for visualization (*p* < 0.05) (Figure 3A). The enrichment results showed that the top-ranked terms were predominantly associated with immune response; among them, the biological processes were mainly enriched in negative regulation of apoptotic process, negative regulation of gene expression, protein phosphorylation, and positive regulation of I-κB kinase/NF-κB signaling. The cellular components were primarily involved in the extracellular region and the cyclin B1-CDK1 complex, and molecular functions were primarily involved in histone kinase activity, protein serine/threonine kinase activity, and low-density lipoprotein receptor activity. For the KEGG pathway analysis, a total of 66 significantly enriched pathways (*p* < 0.05) were identified, with the top 20 pathways presented in Figure 3B. The analysis revealed that the targets were significantly enriched in pathways related to lipids and atherosclerosis, as well as FoxO, p53, and MAPK signaling pathways. Collectively, these enrichment results indicate that the core targets are primarily involved in β-catenin, suggesting that berberine possibly modulates β-catenin pathways and repairs intercellular tight junctions, thereby alleviating *G. parasuis*-induced meningitis.

### 3.3. Bacterial Growth Curve and hBMECs Infected with G. parasuis Analysis

**Figure 4:**
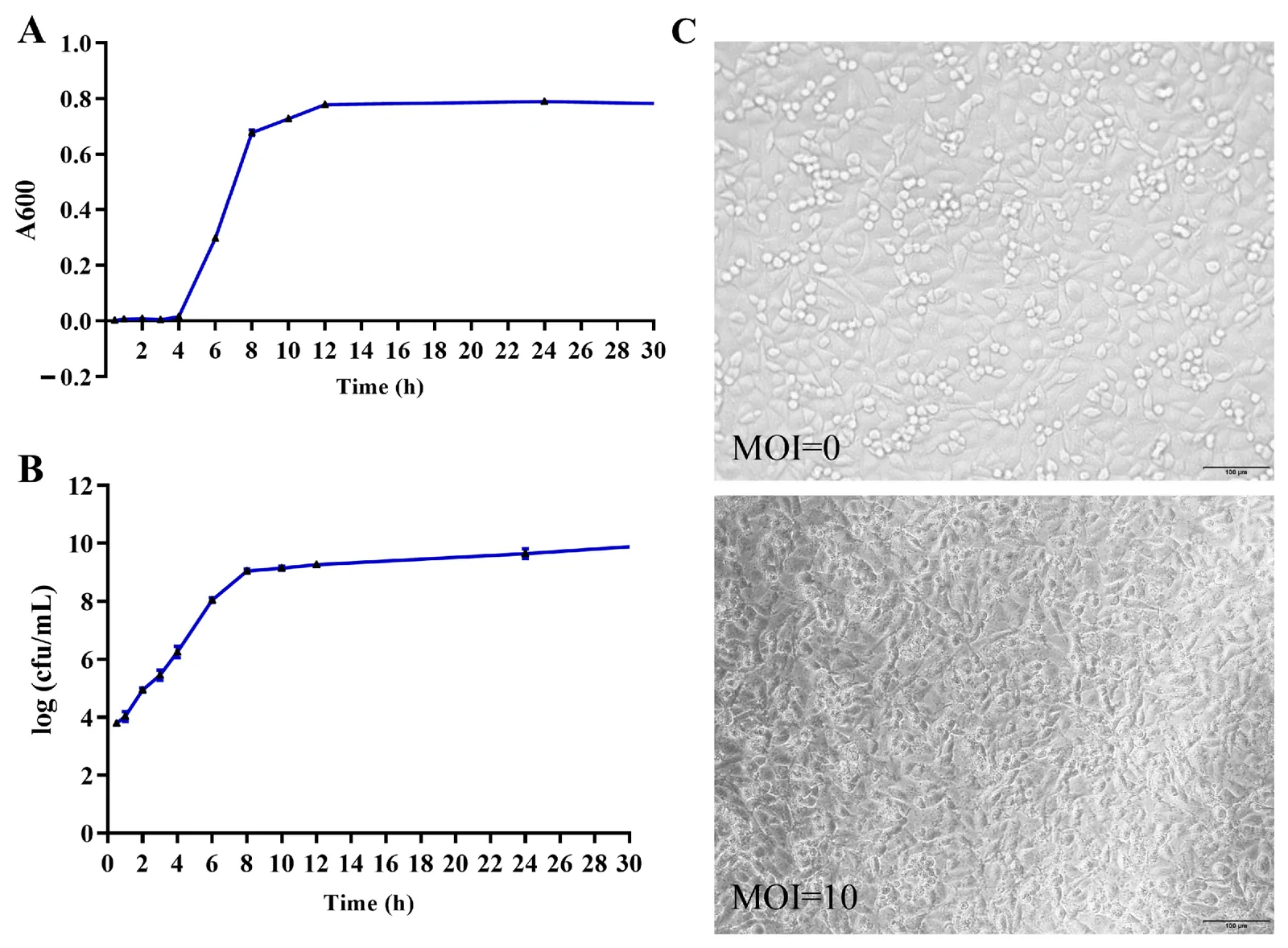
Bacterial growth curves and MOI outcomes for bacteria-infected hBMECs. (**A**) Growth curve data based on absorbance readings. (**B**) Growth curve data obtained by the spread-plate technique. (**C**) MOI results for bacterial infection of hBMECs.

As shown in Figure 4A,B, the growth of *G. parasuis* is in the logarithmic phase at 4–8 h and in the stable phase at 8–24 h. Therefore, we chose 8 h as the growth time of *G. parasuis*. Figure 4C shows the cell-bacteria co-culture assay. Significant morphological changes in hBMECs occurred when the complex of infection (MOI) was equal to 10. The cells did not show massive cell death. Then we chose MOI = 10 for the follow-up test.

### 3.4. Cytotoxic Effects of Berberine in hBMECs 

**Figure 5:**
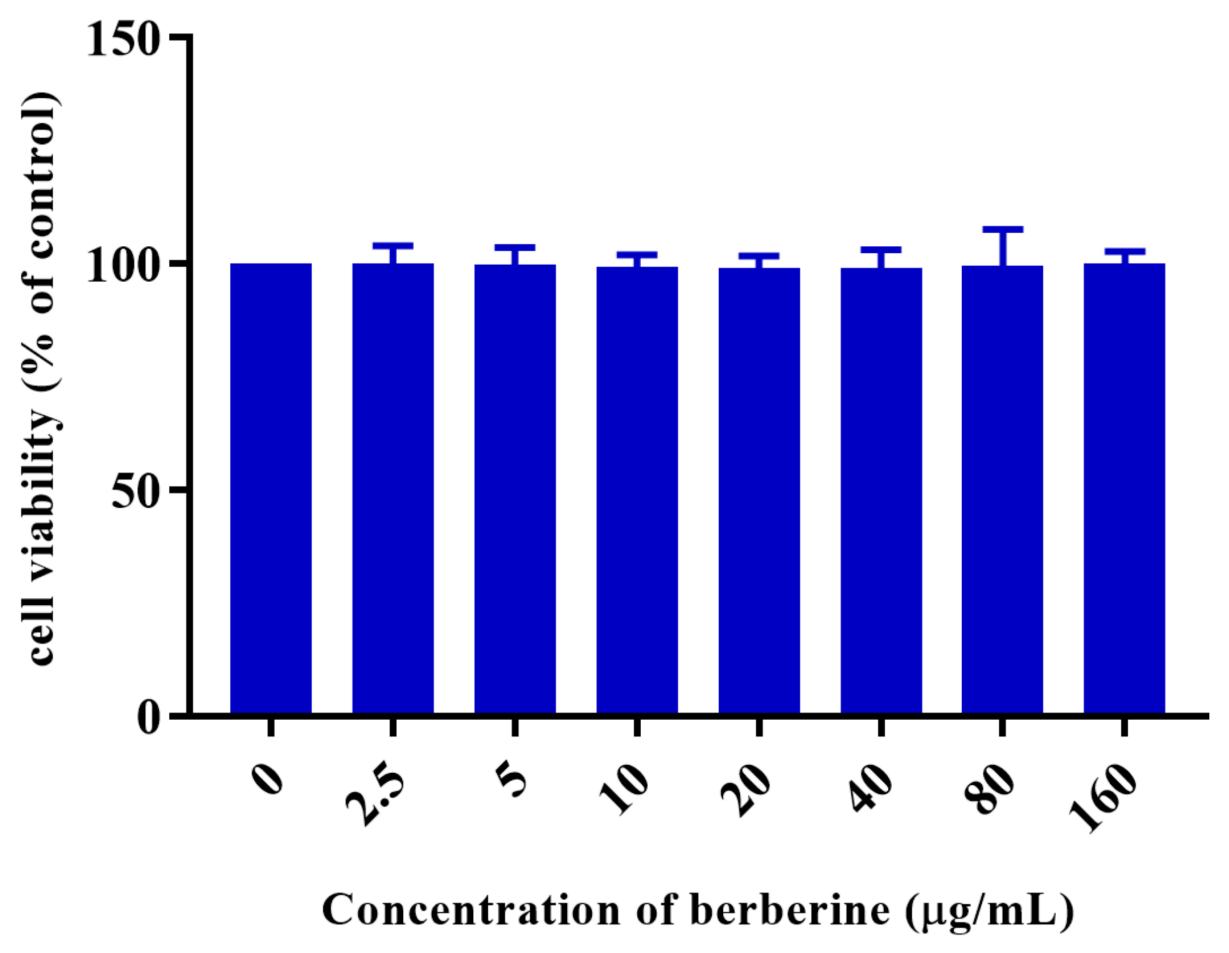
Results of berberine cytotoxicity in hBMECs.

The cytotoxicity of berberine was evaluated by assessing cell viability using the CCK-8 assay. As shown in Figure 5, berberine concentrations below 160 μg/mL did not cause significant changes in the viability of hBMECs. Based on these findings, concentrations of 2.5, 10, and 20 μg/mL were selected as the working concentrations for subsequent experiments.

### 3.5. Validation of the Interaction Between Berberine and β-Catenin Protein

**Figure 6:**
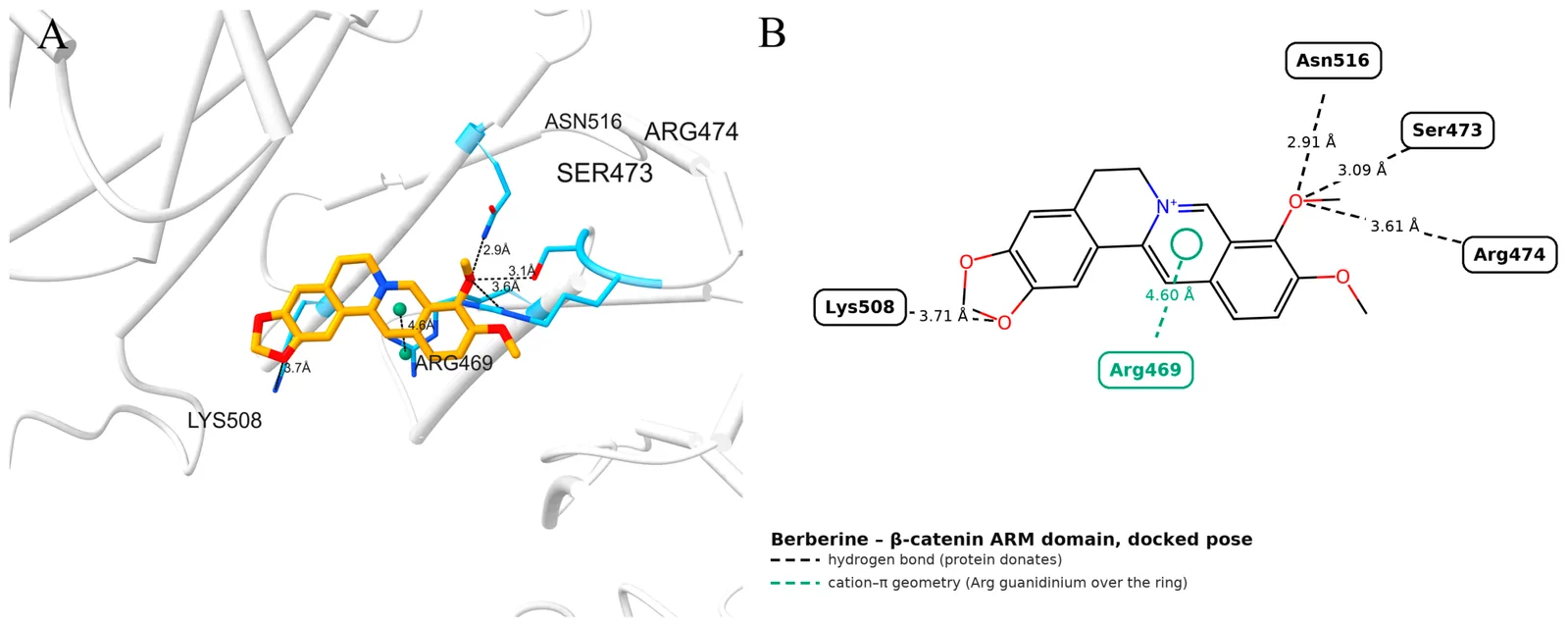
The results of molecular docking. (**A**) The results shown as 3D (docking score = 4.17). (**B**) The results shown as 2D.

**Figure 7:**
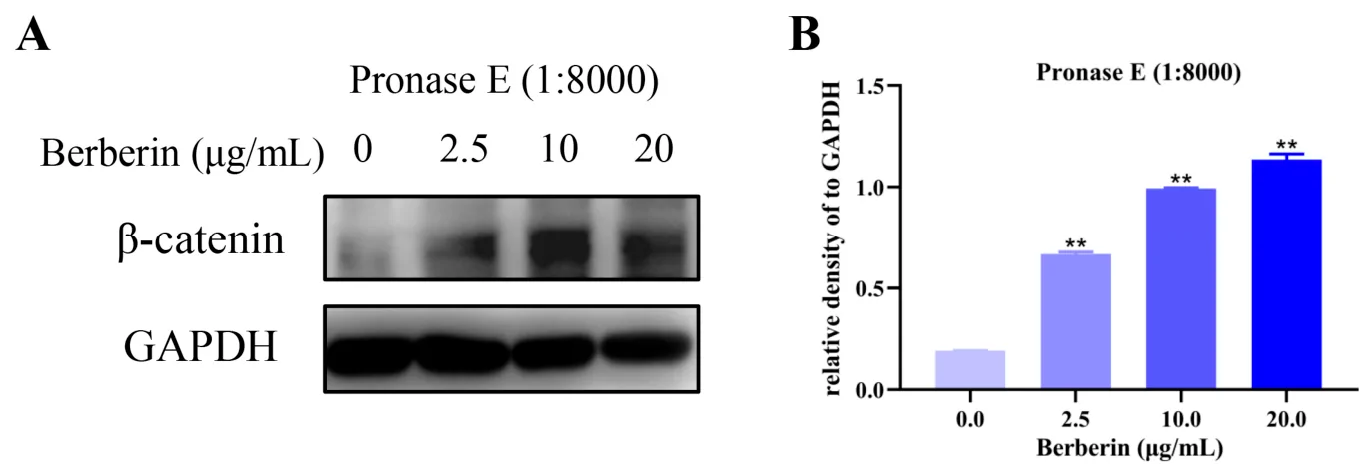
DARTS analysis of the binding ability of berberine to CTNNB1. (**A**) The results of western blot. (**B**) The results of Western blot quantification (** = *p* < 0.01).

To investigate the interaction between the protein berberine and β-catenin protein, molecular docking combined with the DARTS assay was employed. The molecular docking results (Figure 6A,B) showed that berberine binds to the target β-catenin via hydrogen bonds involving amino acid residues at positions Arg474, Lyl508, Asn516, and Ser473, and cation-π geometry at position Arg469 (docking score = −7.52 kcal/mol). DARTS analysis was performed using 2.5, 10, and 20 μg/mL berberine, and the results (Figure 7) demonstrated that, compared with the DMSO group, the β-catenin protein in the berberine-treated group exhibited enhanced enzymatic stability against pronase digestion (*p* < 0.01), indicating that berberine can bind to the β-catenin protein and thereby increase its resistance to proteolysis. This suggests that berberine could bind to β-catenin and enhance the enzymatic stability of the β-catenin protein, further validating the molecular docking findings.

### 3.6. G. parasuis Disrupts the Junctions Between Cells, and Berberine Alleviates the Damage to Cells by G. parasuis

**Figure 8:**
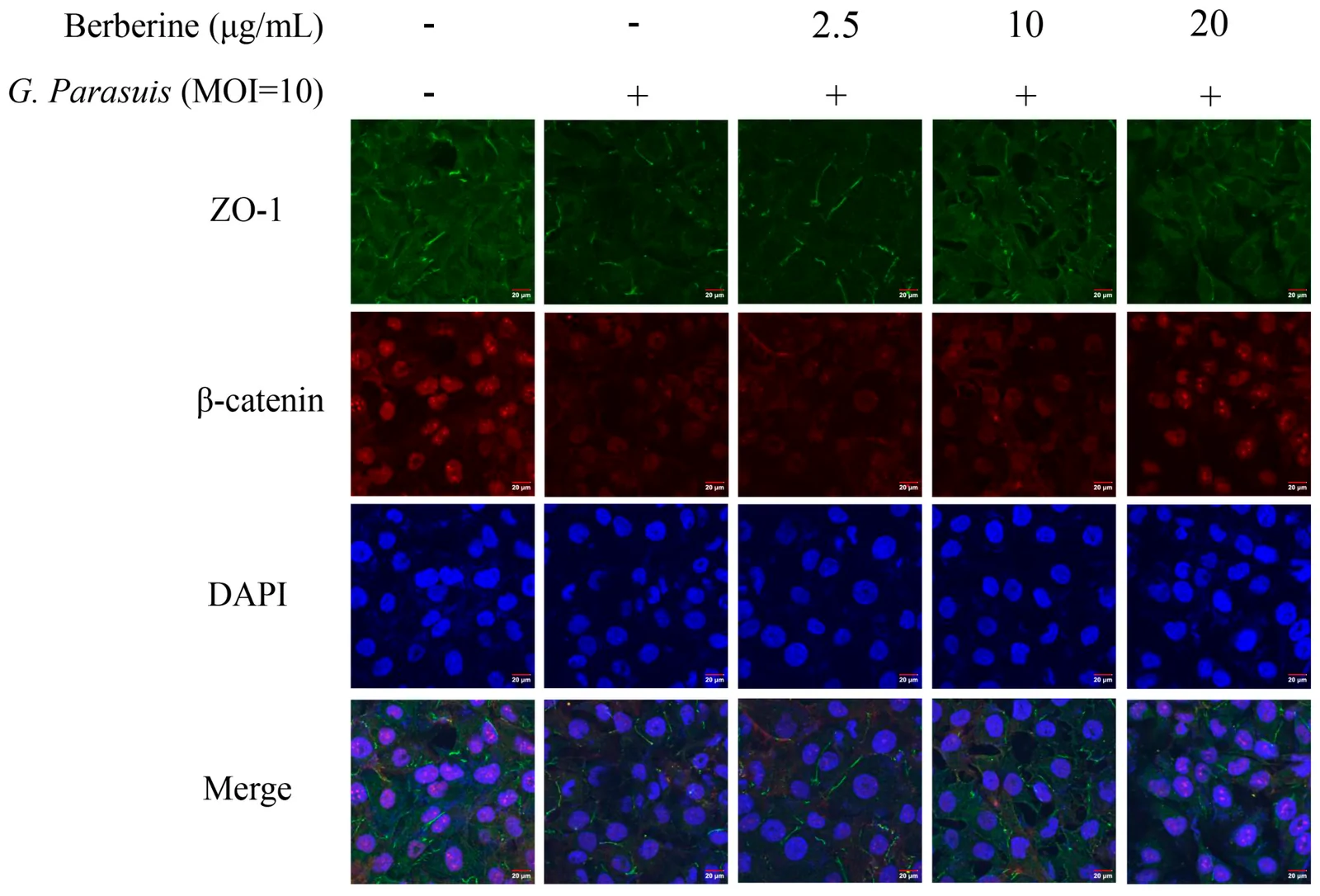
Indirect immunofluorescence detection of the expression level of CTNNB1 and ZO-1 in hBMECs(scale bar = 20 μm). Cells were pretreated with berberine followed by infection with *G. parasuis* (Green fluorescence: ZO-1; red fluorescence: CTNNB1; blue fluorescence: nuclei (DAPI); Merge: co-localization).

To further investigate the mechanism of meningitis induced by *G. parasuis* and the protective effects of berberine, an indirect immunofluorescence assay was performed in this study. The expression and localization of β-catenin, a core protein identified by network pharmacology, and ZO-1, a representative protein of tight junctions, were examined. The results showed that after infection of hBMECs with *G. parasuis*, the protein expression levels of both β-catenin and ZO-1 were significantly downregulated (Figure 8). β-catenin was predominantly localized in the cytoplasm, while ZO-1 was mainly distributed at the cell membrane, suggesting that *G. parasuis* may disrupt tight junctions and adherens junctions, thereby contributing to the pathogenesis of meningitis. Furthermore, berberine treatment resulted in a concentration-dependent upregulation of β-catenin and ZO-1 expression, suggesting that berberine may exert its protective effect against *G. parasuis*-induced meningitis by inhibiting the aberrant downregulation of these proteins and preserving the integrity of cell junctions.

## 4. Discussion

As the primary causative agent of swine Glässer’s disease, *G. parasuis* causes meningitis as one of the most severe clinical manifestations, leading to significant economic losses in the swine industry worldwide [6,28]. Despite the substantial burden of this disease, studies on the molecular mechanisms underlying *G. parasuis*-induced meningitis remain scarce, particularly those involving BBB disruption and junctional protein regulation. Moreover, targeted therapeutic strategies for *G. parasuis* meningitis are currently lacking. Thus, we employed a network pharmacology-based strategy combined with in vitro experimental validation and identified CTNNB1 (encodes the β-catenin protein) as a key target of berberine in alleviating *G. parasuis* meningitis. Our results demonstrated that berberine effectively reverses *G. parasuis*-induced downregulation of ZO-1 and β-catenin in hBMECs, thereby restoring intercellular junctional integrity and preserving BBB function. Collectively, these findings reveal a novel mechanism by which berberine exerts its protective effects against *G. parasuis* meningitis, and provide a mechanistic basis for the development of berberine as a promising alternative therapeutic agent against this disease.

Through the construction of a PPI network, network pharmacology identified CTNNB1 as a core target among multiple candidate targets, suggesting that this target may play a crucial role in the interaction network underlying the anti-*G. parasuis* effects of berberine. CTNNB1 encodes β-catenin, a multifunctional hub coordinating cadherin-based cell adhesion and canonical Wnt signaling [29]. As an adhesion molecule, β-catenin links the cytoplasmic tail of cadherins (E-cadherin in epithelia, VE-cadherin in endothelium) to the actin cytoskeleton, stabilizing adherens junctions and maintaining barrier integrity [30,31]. This adhesive function is especially critical in the BBB, where β-catenin-containing adherens junctions, together with tight-junction proteins such as ZO-1, claudin-5 and occludin, establish and maintain the continuous, impermeable cerebral endothelium [4,5]; FoxM1-dependent regulation of β-catenin has been shown to be required for endothelial junction re-annealing and barrier repair [32]. Given its essential role in BBB integrity, β-catenin dysregulation has been implicated in a range of central nervous system disorders, including neurodevelopmental diseases, stroke, and infectious meningitis [33,34,35]. Despite the established link between *G. parasuis* and Wnt/β-catenin pathway activation [36], the contribution of β-catenin to *G. parasuis* meningitis, particularly its potential as an actionable target for intervention, has not been investigated. In this study, we demonstrate that β-catenin serves as a direct target of berberine, and that berberine treatment effectively reverses *G. parasuis*-induced downregulation of β-catenin, thereby restoring BBB junctional integrity. These findings not only extend the known functions of β-catenin from neurodevelopmental and vascular contexts to bacterial meningitis, but also identify CTNNB1 as a promising therapeutic target for berberine in the treatment of this disease.

ZO-1 is a principal scaffolding protein of the tight junction complex, which anchors transmembrane proteins to the actin cytoskeleton and plays a pivotal role in maintaining the permeability of the BBB [37]. Downregulation of ZO-1 has been shown to disrupt tight junction integrity and increase BBB paracellular permeability [38,39]. In experimental subarachnoid hemorrhage, BBB disruption was closely associated with the degradation of ZO-1 [40]. Consistently, in lipopolysaccharide-challenged BBB models, downregulation of miR-130a-5p restored ZO-1 expression and elevated trans endothelial electrical resistance, thereby suppressing BBB leakage and indicating that ZO-1 depletion impairs tight junction barrier function and promotes paracellular flux [41]. Collectively, these observations establish that diminished ZO-1 expression destabilizes tight junctions and promotes BBB hyperpermeability. In the present study, we found that *G. parasuis* infection significantly downregulated the expression of both ZO-1 and β-catenin, suggesting that this pathogen may compromise BBB integrity by synergistically disrupting tight junctions and adherens junctions, thus enabling its translocation across the BBB into the central nervous system. Notably, although ZO-1 was not predicted as a direct target of berberine by network pharmacology, it was subsequently employed as a functional verification indicator to comprehensively evaluate the protective effects of berberine on BBB junctional complexes. As a tight junction scaffolding protein, ZO-1 cooperates with β-catenin to maintain the structural and functional integrity of the BBB. Therefore, changes in ZO-1 expression levels can objectively reflect the restorative efficacy of berberine on the junctional complex.

Previous studies have demonstrated that *G. parasuis* infection elicits a robust host inflammatory response, with activation of the MAPK and NF-κB signaling pathways that subsequently disrupt the junctional complex by promoting degradation of junctional proteins or suppressing their gene transcription [42]. In our network pharmacology-based screening, β-catenin was predicted as a direct target of berberine. Further GO and KEGG enrichment analyses revealed that the potential targets of berberine are significantly associated with biological processes and pathways closely related to the infection-induced pathology, including positive regulation of I-κB kinase/NF-κB signaling, protein phosphorylation, and negative regulation of the apoptotic process, as well as the FoxO, p53, and MAPK signaling pathways. These results not only corroborate the involvement of the classic inflammatory cascades (MAPK/NF-κB) in the disease context, but also suggest that berberine may concurrently modulate cell survival and stress-response pathways (FoxO/p53) that are known to crosstalk with junctional stability and endothelial integrity. Taken together, the integrative pharmacology data support a multifaceted regulatory role of berberine in counteracting the detrimental effects of *G. parasuis* infection.

Mechanistically, as a natural alkaloid with broad-spectrum anti-inflammatory activity, berberine may restore normal expression levels of ZO-1 and β-catenin by inhibiting the aberrant activation of these signaling pathways, particularly the MAPK and NF-κB axes. Furthermore, given that β-catenin was identified as a direct target, the molecular docking and DARTS results suggest that berberine may directly bind to β-catenin, enhancing its stability at the plasma membrane and thereby synergistically preserving the normal structure and function of the ZO-1-containing tight junction complex. The observed downregulation of ZO-1 expression following berberine treatment may be partially attributable to a compensatory or cooperative effect secondary to the functional restoration of β-catenin, reinforcing the notion that berberine protects BBB integrity through coordinated modulation of both tight and adherens junction components. Moreover, CCK-8 data confirm that berberine is devoid of direct cytotoxicity in porcine endothelial cells up to 160 μg/mL, providing a safety benchmark for future exploration of alternative administration routes that could bypass the first-pass effect. Although berberine is generally considered to have low toxicity in animals, and its pharmacokinetic properties and therapeutic potentials have been comprehensively reviewed in diverse vascular diseases, these findings cannot be directly extrapolated to Glässer’s-disease-affected pigs [43,44]. Therefore, future studies evaluating berberine’s pharmacokinetics, efficacy, and safety in a porcine model of Glässer’s disease are required before clinical application.

Overall, this study provides a valuable contribution by identifying β-catenin (encoded by CTNNB1) as a key target of berberine against *G. parasuis*-induced meningitis and by revealing its association with inflammatory (MAPK/NF-κB) and stress response (FoxO/p53) pathways, thereby offering a mechanistic basis for the potential repurposing of berberine as an antibiotic alternative for Glässer’s disease. Future studies should confirm the precise binding mode between berberine and β-catenin using SPR, ITC, or co-crystallization, and clarify the causal relationship between β-catenin and ZO-1 through gene silencing or overexpression experiments. The cross-regulatory network among other core targets (e.g., MAPK14, CASP3) and β-catenin also warrants further investigation using co-immunoprecipitation or pathway-specific inhibitor assays. Importantly, in vivo validation in animal models, particularly mice and pigs, is needed to confirm the protective effects observed in vitro and to evaluate translational potential. As the activity and toxicity of berberine in pharmacokinetic and clinical studies remain unknown for pigs, including those with illness such as Glässer’s disease, the development of a standardized dosage, administration route, duration of action, and assessment of adverse reactions of berberine in pigs are still lacking. Systematic pharmacokinetic and toxicological evaluations in pigs, together with optimization of the dosing regimen, will therefore be essential to facilitate the clinical application of berberine for the treatment of *G. parasuis* meningitis.

## 5. Conclusions

In conclusion, our findings demonstrate that β-catenin is a key target of berberine in alleviating *G. parasuis*-induced meningitis. We reveal that berberine exerts its protective effects by targeting β-catenin to restore intercellular junctional integrity, thereby preserving BBB function. This study not only provides novel insights into the pathogenesis of *G. parasuis* meningitis from the perspective of junctional protein dysregulation, but also establishes a mechanistic foundation for the development of berberine as a promising candidate therapeutic agent against this disease.

## Data Availability

All data in this article are presented in the article.

## Abbreviations

The following abbreviations are used in this manuscript:

3D	three-dimensional
BBB	Blood–brain barrier
CTNNB1	Catenin (Cadherin-Associated Protein), Beta 1
DARTS	Drug Affinity Responsive Target Stability
FBS	Fetal Bovine Serum
FoxO	FoxO
*G. parasuis*	*Glaesserella parasuis*
GO	Gene Ontology
hBMECs	human brain microvascular endothelial cells
ITC	isothermal titration calorimetry
I-κB	inhibitor of NF-κB
KEGG	Kyoto Encyclopedia of Genes and Genomes
MAPK	mitogen-associated protein kinase
MIC	minimum inhibitory concentration
NAD+	Nicotinamide adenine dinucleotide
NF-κB	Nuclear Factor kappa-light-chain-enhancer of activated B
OD	Nicotinamide adenine dinucleotide
p53	Tumor Protein 53
PLIP	Protein-ligand interaction profiler
PPI	protein–protein interaction
SPR	surface plasmon resonance
TSB	Tryptic Soy Broth

## References

[1] Alvarez-Vega L., De La Cruz-Monroy K.S.M., Garcia-Barraza A., Carhuaricra D., Llaja-Bernedo J.L., Huamancha-Pulido L.L., Medina-Gonzales A., Sedano-Sanchez A.F. (2025). Genomic insights into diversity, antimicrobial resistance and virulence of *Glaesserella parasuis* from diseased swine in Peru. Front. Microbiol..

[2] Ni H.B., Gong Q.L., Zhao Q., Li X.Y., Zhang X.X. (2020). Prevalence of *Haemophilus parasuis*“*Glaesserella parasuis*” in pigs in China: A systematic review and meta-analysis. Prev. Vet. Med..

[3] Dai K., He L., Chang Y.F., Cao S., Zhao Q., Huang X., Wu R., Huang Y., Yan Q., Han X. (2018). Basic Characterization of Natural Transformation in a Highly Transformable Haemophilus parasuis Strain SC1401. Front. Cell Infect. Microbiol..

[4] Huang J., Wang X., Cao Q., Feng F., Xu X., Cai X. (2016). ClpP participates in stress tolerance and negatively regulates biofilm formation in *Haemophilus parasuis*. Vet. Microbiol..

[5] Zhou Q., Feng S., Zhang J., Jia A., Yang K., Xing K., Liao M., Fan H. (2016). Two Glycosyltransferase Genes of *Haemophilus parasuis* SC096 Implicated in Lipooligosaccharide Biosynthesis, Serum Resistance, Adherence, and Invasion. Front. Cell Infect. Microbiol..

[6] Guo L., Cheng H., Fu S., Liu J., Zhang Y., Qiu Y., Chen H. (2022). Methylome and Transcriptome-Based Integration Analysis Identified Molecular Signatures Associated With Meningitis Induced by *Glaesserella parasuis*. Front. Immunol..

[7] Sun P., Yang Y., Yang L., Qian Y., Liang M., Chen H., Zhang J., Qiu Y., Guo L., Fu S. (2024). Quercetin Protects Blood-Brain Barrier Integrity via the PI3K/Akt/Erk Signaling Pathway in a Mouse Model of Meningitis Induced by *Glaesserella parasuis*. Biomolecules.

[8] Zhu Y., Guan L., Zhang J., Xue Y., Zhao Z. (2024). Research progress into the principles and methods underlying capsular typing of *Glaesserella parasuis*. Vet. Res..

[9] de la Fuente A.J., Tucker A.W., Navas J., Blanco M., Morris S.J., Gutiérrez-Martín C.B. (2007). Antimicrobial susceptibility patterns of *Haemophilus parasuis* from pigs in the United Kingdom and Spain. Vet. Microbiol..

[10] Fei Z., Yin W., Li-Rui L., Hai-Yan H., Xue-Ping Y. (2012). Isolation and Identification of *Haemophilus parasuis* and Establishment of its Detection Method of Indirect ELISA. J. Anim. Vet. Adv..

[11] Costa-Hurtado M., Barba-Vidal E., Maldonado J., Aragon V. (2020). Update on Glässer’s disease: How to control the disease under restrictive use of antimicrobials. Vet. Microbiol..

[12] Duan Y., Hao Y., Feng H., Shu J., He Y. (2025). Research progress on *Haemophilus parasuis* vaccines. Front. Vet. Sci..

[13] Guo P., Liu X., Li X., Ihsan A., Wu Z., Fu S., Ye C., Qiu Y., Wang X., Lu Q. (2025). Protective Effects of Luteolin on Glaesserella parasuis-Induced Injury: An In vitro Study with Porcine Vascular Endothelial Cells. Antibiotics.

[14] Archie S.R., Al Shoyaib A., Cucullo L. (2021). Blood-Brain Barrier Dysfunction in CNS Disorders and Putative Therapeutic Targets: An Overview. Pharmaceutics.

[15] Senthilnathan S., Nallusamy G., Varadaraj P., Reddy K.S.S., Ravipati C. (2024). Cortical Laminar Necrosis as a Rare Complication of Streptococcus pneumoniae Meningitis: A Case Report. Cureus.

[16] Yang R.C., Qu X.Y., Xiao S.Y., Li L., Xu B.J., Fu J.Y., Lv Y.J., Amjad N., Tan C., Kim K.S. (2019). Meningitic Escherichia coli-induced upregulation of PDGF-B and ICAM-1 aggravates blood-brain barrier disruption and neuroinflammatory response. J. Neuroinflamm..

[17] Hou Q., He W.J., Wu Y.S., Hao H.J., Xie X.Y., Fu X.B. (2020). Berberine: A Traditional Natural Product With Novel Biological Activities. Altern. Ther. Health Med..

[18] Peng L., Kang S., Yin Z., Jia R., Song X., Li L., Li Z., Zou Y., Liang X., Li L. (2015). Antibacterial activity and mechanism of berberine against *Streptococcus agalactiae*. Int. J. Clin. Exp. Pathol..

[19] Ding W., Gu Q., Liu M., Zou J., Sun J., Zhu J. (2023). Astrocytes-derived exosomes pre-treated by berberine inhibit neuroinflammation after stroke via miR-182-5p/Rac1 pathway. Int. Immunopharmacol..

[20] Liu W., Wei R., Xu J., Liu Z., Li Y. (2025). Inflammation-related biomarkers and berberine therapy in post-stroke depression: Evidence from bioinformatics, machine learning, and experimental validation. Front. Neurosci..

[21] Qin Z., Shi D.D., Li W., Cheng D., Zhang Y.D., Zhang S., Tsoi B., Zhao J., Wang Z., Zhang Z.J. (2023). Berberine ameliorates depression-like behaviors in mice via inhibiting NLRP3 inflammasome-mediated neuroinflammation and preventing neuroplasticity disruption. J. Neuroinflamm..

[22] Sun C., Sha S., Shan Y., Gao X., Li L., Xing C., Guo Z., Du H. (2025). Intranasal Delivery of BACE1 siRNA and Berberine via Engineered Stem Cell Exosomes for the Treatment of Alzheimer’s Disease. Int. J. Nanomed..

[23] Guo P., Xue Y., Zhang D., Lu Q., Liu Y., Xiong J., Ye C., Fu S., Wu Z., Wang X. (2025). Network Pharmacology to Unveil the Mechanism of Berberine in the Treatment of Streptococcus suis Meningitis in Humans and Pigs. Toxics.

[24] Jia Y., Hao C., Yang Q., Zhang W., Li G., Liu S., Hua X. (2021). Inhibition of *Haemophilus parasuis* by berberine and proteomic studies of its mechanism of action. Res. Vet. Sci..

[25] Guo P., Li X., Xue Y., Lu Q., Liu Y., Xiong J., Wu Z., Fu S., Ye C., Wang X. (2024). Using network pharmacology and molecular docking to uncover the mechanism by which quercetin alleviates deoxynivalenol-induced porcine intestinal injury. Toxicon.

[26] Schake P., Bolz S.N., Linnemann K., Schroeder M. (2025). PLIP 2025: Introducing protein-protein interactions to the protein-ligand interaction profiler. Nucleic Acids Res..

[27] Geng J., Liu W., Gao J., Jiang C., Fan T., Sun Y., Qin Z.H., Xu Q., Guo W., Gao J. (2019). Andrographolide alleviates Parkinsonism in MPTP-PD mice via targeting mitochondrial fission mediated by dynamin-related protein 1. Br. J. Pharmacol..

[28] Guo P., Fan J., Dong Y., Zhang J., Lu Q., Ye C., Fu S., Wu Z., Liu Y., Qiu Y. (2026). *Glaesserella parasuis* Infection Modulates the Transcriptome of Porcine Peritoneal Mesothelial Primary Cells: Implications for Understanding Peritoneal Invasion Mechanisms. Biology.

[29] van der Wal T., van Amerongen R. (2020). Walking the tight wire between cell adhesion and WNT signalling: A balancing act for β-catenin. Open Biol..

[30] Pećina-Slaus N., Zigmund M., Kusec V., Martić T.N., Cacić M., Slaus M. (2007). E-cadherin and beta-catenin expression patterns in malignant melanoma assessed by image analysis. J. Cutan. Pathol..

[31] Mège R.M., Ishiyama N. (2017). Integration of Cadherin Adhesion and Cytoskeleton at Adherens Junctions. Cold Spring Harb. Perspect. Biol..

[32] Kim Y.E., Kim M., Kim S., Lee R., Ujihara Y., Marquez-Wilkins E.M., Jiang Y.H., Yang E., Kim H., Lee C. (2025). Endothelial SHANK3 regulates tight junctions in the neonatal mouse blood-brain barrier through β-Catenin signaling. Nat. Commun..

[33] Zhuang W., Ye T., Wang W., Song W., Tan T. (2023). CTNNB1 in neurodevelopmental disorders. Front. Psychiatry.

[34] Jiang A., Zhang H., Jia X., Zhao H., Zhao H., Lu Z. (2025). Identification of PANoptosis-related genes as biomarkers in ischemic stroke. Front. Neurol..

[35] Krishnan S., Fernandez G.E., Sacks D.B., Prasadarao N.V. (2012). IQGAP1 mediates the disruption of adherens junctions to promote Escherichia coli K1 invasion of brain endothelial cells. Cell Microbiol..

[36] Hua K., Gong H., Xu Q., Li T., Ma B., Li Y., He R., Bi D., Zhou R., Luo R. (2020). P38 mitogen-activated protein kinase promotes Wnt/β-catenin signaling by impeding Dickkofp-1 expression during *Haemophilus parasuis* infection. Cytokine.

[37] Li Y., Zhou F., You J., Gong X. (2024). Annexin A1 Mitigates Blood-Brain Barrier Disruption in a Sepsis-Associated Encephalopathy Model by Enhancing the Expression of Occludin and Zonula Occludens-1 (ZO-1). CNS Neurosci. Ther..

[38] Wu S., Wang J., Liu J., Zhu H., Li R., Wan X., Lei J., Li Y., You C., Hu F. (2023). Programmed cell death 10 increased blood-brain barrier permeability through HMGB1/TLR4 mediated downregulation of endothelial ZO-1 in glioblastoma. Cell Signal..

[39] Guo P., Lu Q., Hu S., Yang Y., Wang X., Yang X., Wang X. (2023). Daucosterol confers protection against T-2 toxin induced blood-brain barrier toxicity through the PGC-1α-mediated defensive response in vitro and in vivo. J. Hazard. Mater..

[40] Sun X.G., Duan H., Jing G., Wang G., Hou Y., Zhang M. (2019). Inhibition of TREM-1 attenuates early brain injury after subarachnoid hemorrhage via downregulation of p38MAPK/MMP-9 and preservation of ZO-1. Neuroscience.

[41] Gan N., Zhou Y., Li J., Wang A., Cao Y. (2024). Propofol Suppresses LPS-induced BBB Damage by Regulating miR-130a-5p/ZO-1 Axis. Mol. Biotechnol..

[42] Hua K., Li Y., Zhou H., Hu X., Chen Y., He R., Luo R., Zhou R., Bi D., Jin H. (2018). *Haemophilus parasuis* Infection Disrupts Adherens Junctions and Initializes EMT Dependent on Canonical Wnt/β-Catenin Signaling Pathway. Front. Cell Infect. Microbiol..

[43] Meng J., Yu R., Wen Z., Chen S., Xie X., Zhang L., Sun P., Cao X. (2025). Pharmacokinetics of colistin sulfate and berberine sulfate following intramuscular injection as single agents or in combination in swine. One Health Adv..

[44] Mouchtoglou C., Cherlet M., Dehau T., Aluwe M., Ducatelle R., Goossens E., Croubels S., Van Immerseel F. (2025). A low dose of berberine is metabolized in weaned piglets without major changes to gut morphology or gut microbiota. Animals.

